# Intractable Nausea in a Heart Transplant Patient

**DOI:** 10.7759/cureus.26804

**Published:** 2022-07-13

**Authors:** Julian Yet Kwong Horman, Michael Schultz

**Affiliations:** 1 Internal Medicine-Pediatrics, Penn State Health Milton S. Hershey Medical Center, Hershey, USA

**Keywords:** immunosuppression, nausea and vomiting, heart transplant, voriconazole toxicity, orthotopic heart transplant

## Abstract

We present a case of a 59-year-old woman who had been recently diagnosed with a cavitary lung nodule and then started on voriconazole; she had been diagnosed with breast cancer about 10 years prior, which had been treated with anthracyclines and subsequent non-ischemic cardiomyopathy, ultimately requiring an orthotopic heart transplant. She presented to the hospital due to nausea and abdominal pain. She was found to have cholelithiasis, without cholecystitis, and was initially discharged with plans for an outpatient cholecystectomy. However, nausea and pain persisted, and hence she was readmitted and had a cholecystectomy but her nausea continued. Further workup revealed an elevated voriconazole level, and her nausea resolved once the voriconazole was discontinued.

## Introduction

The medical treatment of post-orthotopic heart transplant patients has steadily evolved since the first heart transplant was performed at the University of Cape Town by Dr. Christiaan Barnard in 1967. The initial immunosuppressive regimen for the first heart transplant patient consisted of a combination of hydrocortisone, prednisone, azathioprine, and radiation therapy [1]. Since then, the immunosuppressive regimens have changed significantly. Current guidelines recommend that the maintenance immunosuppression include calcineurin inhibitor-based therapy, often with tacrolimus as the mainstay, along with mycophenolate mofetil, sirolimus, or everolimus as tolerated. Corticosteroids are often also used for the maintenance of immunosuppression. Induction therapy is more varied, with the current guidelines recommending the consideration of induction therapy in certain patients with a high risk for acute rejection or those at high risk of renal dysfunction, but the routine use of immunosuppressive induction in all patients is not currently recommended [2].

One of the feared complications of orthotopic heart transplantation is rejection, which necessitates the need for immunosuppression. This risk of rejection must be balanced with the risks associated with chronic immunosuppression, which includes increased risk and severity of infections. This risk of infection was exhibited in the first orthotopic heart transplant patient, who died 18 days after transplantation due to bilateral pneumonia [3].

Infection after an orthotopic heart transplant is common, with one study finding that 81% of patients have at least one infection within 180 days of heart transplantation. Of these infections, 71% are caused by bacteria or fungus. Pneumonia is the most common bacterial and fungal infection, occurring in 52% of cases [4]. Infections in these patients have their own unique set of complexities, often necessitating long and intricate antimicrobial regimens, which bring their own complications. One study found that 29% of patients receiving antifungal therapy experienced some sort of adverse reaction, with amphotericin and voriconazole being the common culprits. The most common adverse drug reactions are infusion reactions, hypokalemia, nephrotoxicity, and hepatotoxicity [5].

## Case presentation

**Figure 1:**
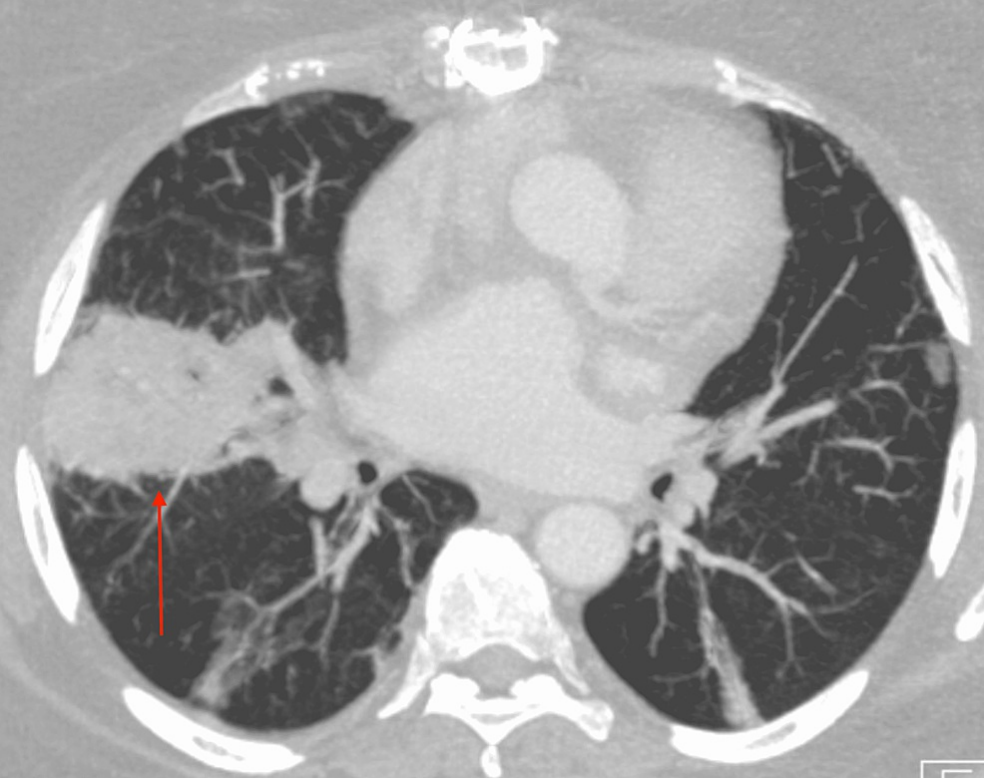
CT thorax The red arrow is pointing at the right middle lobe cavitary lesion CT: computed tomography

The patient was a 59-year-old woman with a history of breast cancer about 10 years prior, which had been treated with anthracyclines leading to non-ischemic cardiomyopathy necessitating an orthotopic heart transplant. About two months after her initial orthotopic heart transplant, a 6.5-cm cavitary and necrotic right middle lobe lung lesion had been found on a thoracic CT, which had been performed due to coughing in the setting of chronic immunosuppression (Figure 1).

She had then been admitted to the hospital for further evaluation, which included a full infectious workup that had only revealed an elevated b-D-glucan. A CT-guided biopsy of the lesion had been performed, which had not grown organisms, but due to the elevated b-D-glucan, she had been started on oral voriconazole 200 mg twice daily for suspected fungal pneumonia.

**Figure 2:**
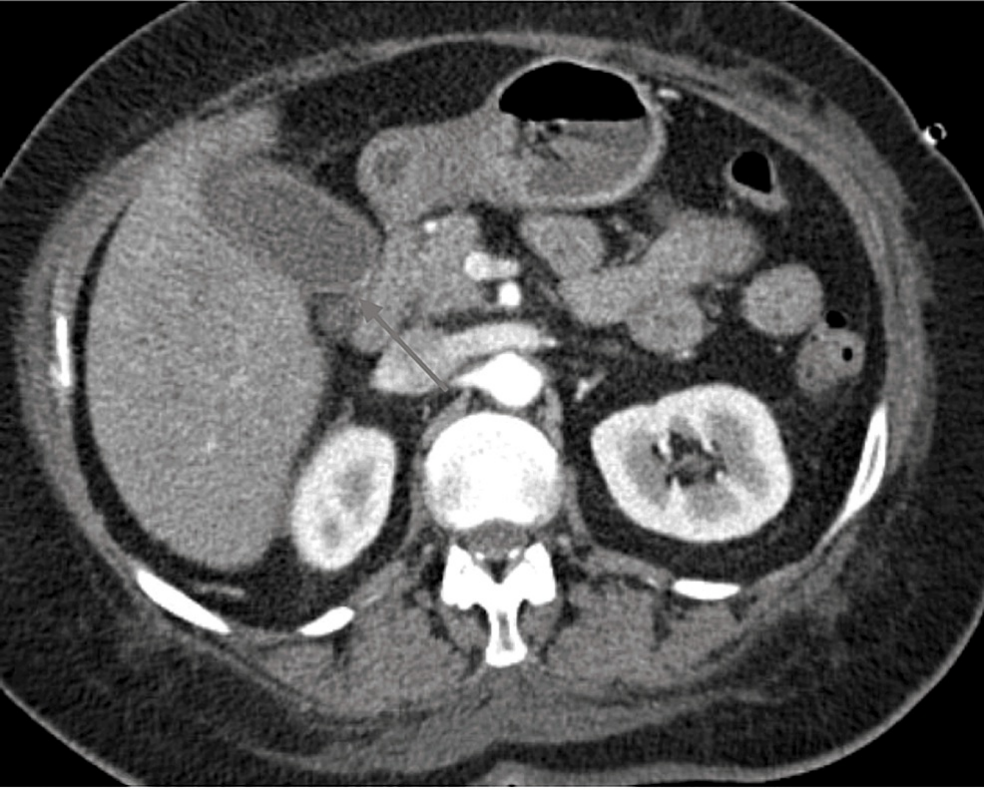
CT of the abdomen Red arrow pointing at the gallbladder with mild surrounding pericholecystic fluid. The gallstones are not seen in this image CT: computed tomography

About two weeks after the fungal pneumonia diagnosis, she presented to the hospital with three days of nausea, right-sided abdominal pain, and weakness. Her initial labs revealed a normal complete blood count, without any leukocytosis. The basic metabolic panel showed hyponatremia with a sodium of 130 mmol/L (normal: 135-145 mmol/L), elevated blood urea nitrogen of 34 mg/dL (normal: 8-25 mg/dL), and elevated creatinine at 2.01 mg/dL (normal 0.45-1.10 mg/dL) with the remainder of the electrolytes returning within normal limits. Her liver function panel was normal, including a normal alanine transferase, aspartate transferase, alkaline phosphatase, total bilirubin, and lipase. A CT of the abdomen and pelvis without contrast was performed due to the abdominal pain, which revealed mild pericholecystic fluid and cholelithiasis but no other findings consistent with acute cholecystitis (Figure 2).

Due to the abdominal pain and the findings of pericholecystic fluid and cholelithiasis on imaging, general surgery was consulted, and they recommended hepatobiliary scintigraphy, which did not show any evidence of cystic duct obstruction. Thus, she was diagnosed with symptomatic cholelithiasis and a surgical follow-up was arranged for a possible outpatient cholecystectomy.

**Figure 3:**
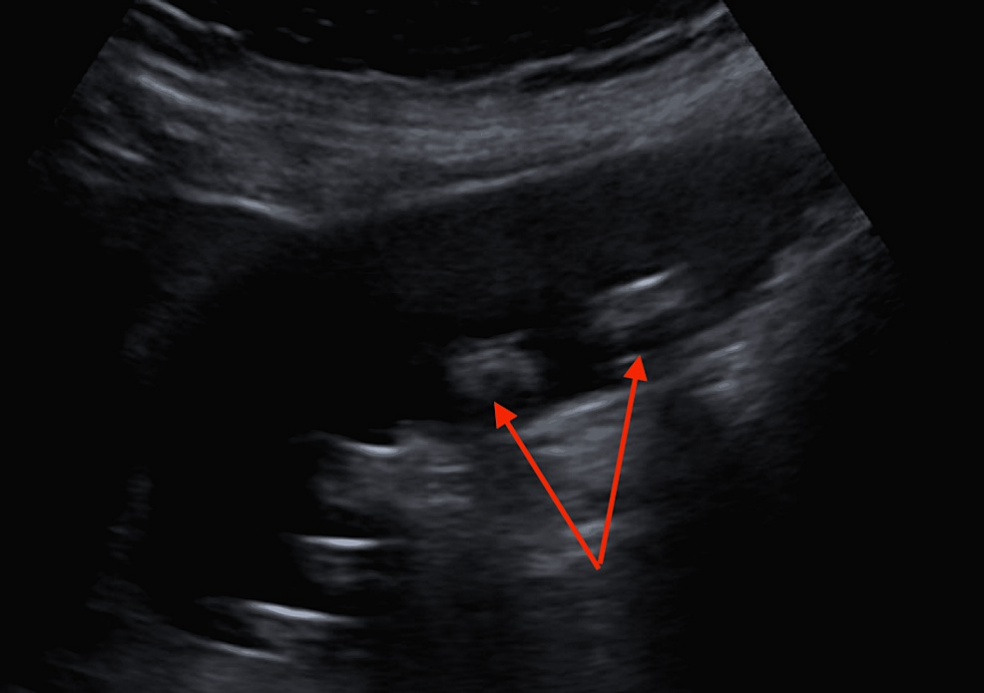
Right upper quadrant ultrasound Red arrows pointing to gallstones within the gallbladder

Three days after being discharged from the hospital, her abdominal pain, nausea, and weakness persisted, and she presented again to the hospital for further evaluation. Her labs were relatively unremarkable. Her complete blood count was stable from the previous, and the basic metabolic panel was all within normal limits with an improvement of her sodium to 138 mmol/L, blood urea nitrogen to 9 mg/dL, and creatinine to 0.83 mg/dL, which was back to her baseline creatinine of 0.6-0.9 mg/dL. Her liver function remained normal as well. An ultrasound of the right upper quadrant was performed, which again showed cholelithiasis without evidence of cholecystitis (Figure 3).

General surgery was consulted for possible cholecystectomy due to recurrent symptomatic cholelithiasis. The next day, she underwent an uneventful laparoscopic cholecystectomy with mild improvement of her abdominal pain, but her nausea and vomiting continued.

**Figure 4:**
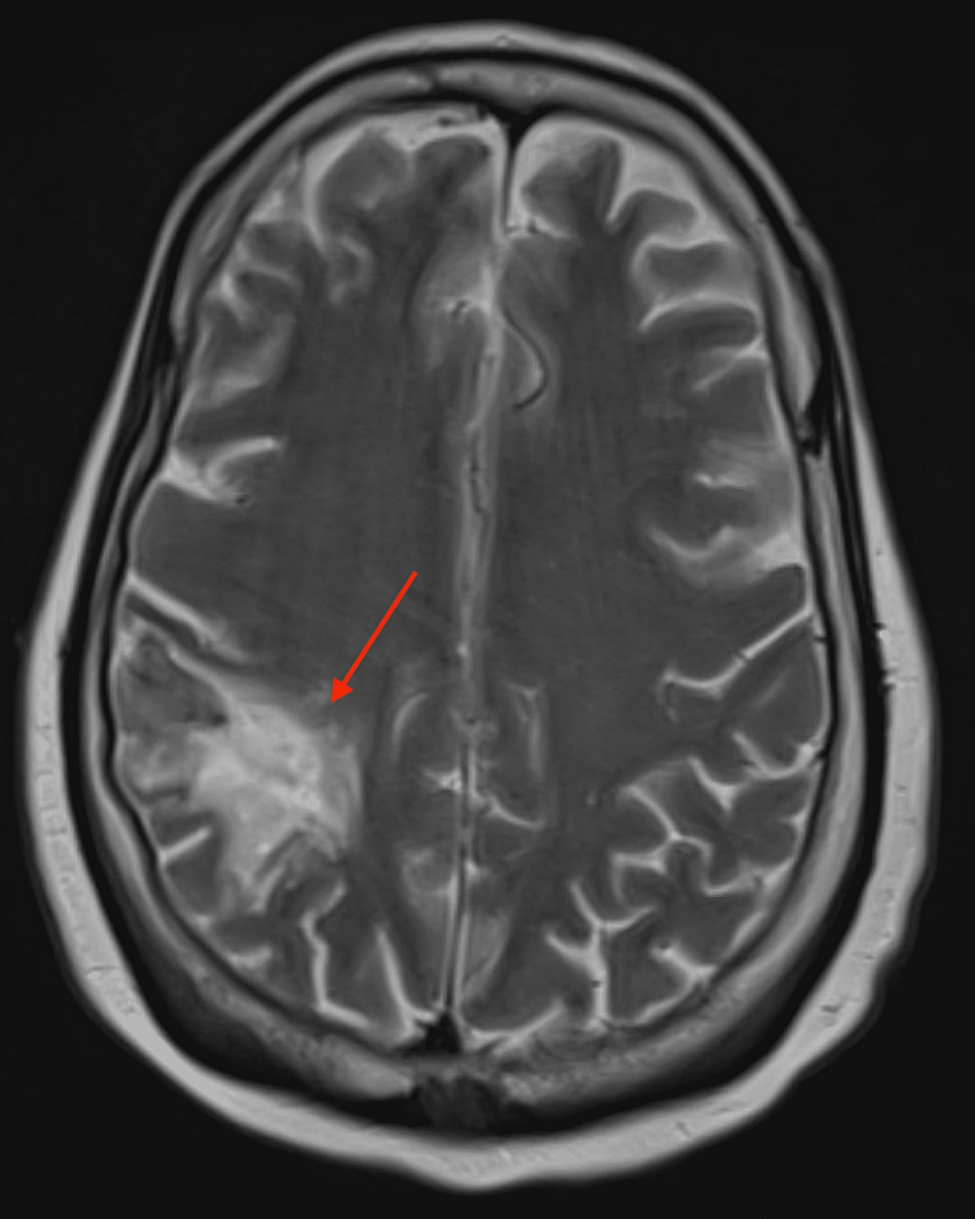
MRI of the brain - image 1 The red arrow is pointing at the chronic right parietal lobe lesion in this T2-weighted MRI image MRI: magnetic resonance imaging

**Figure 5:**
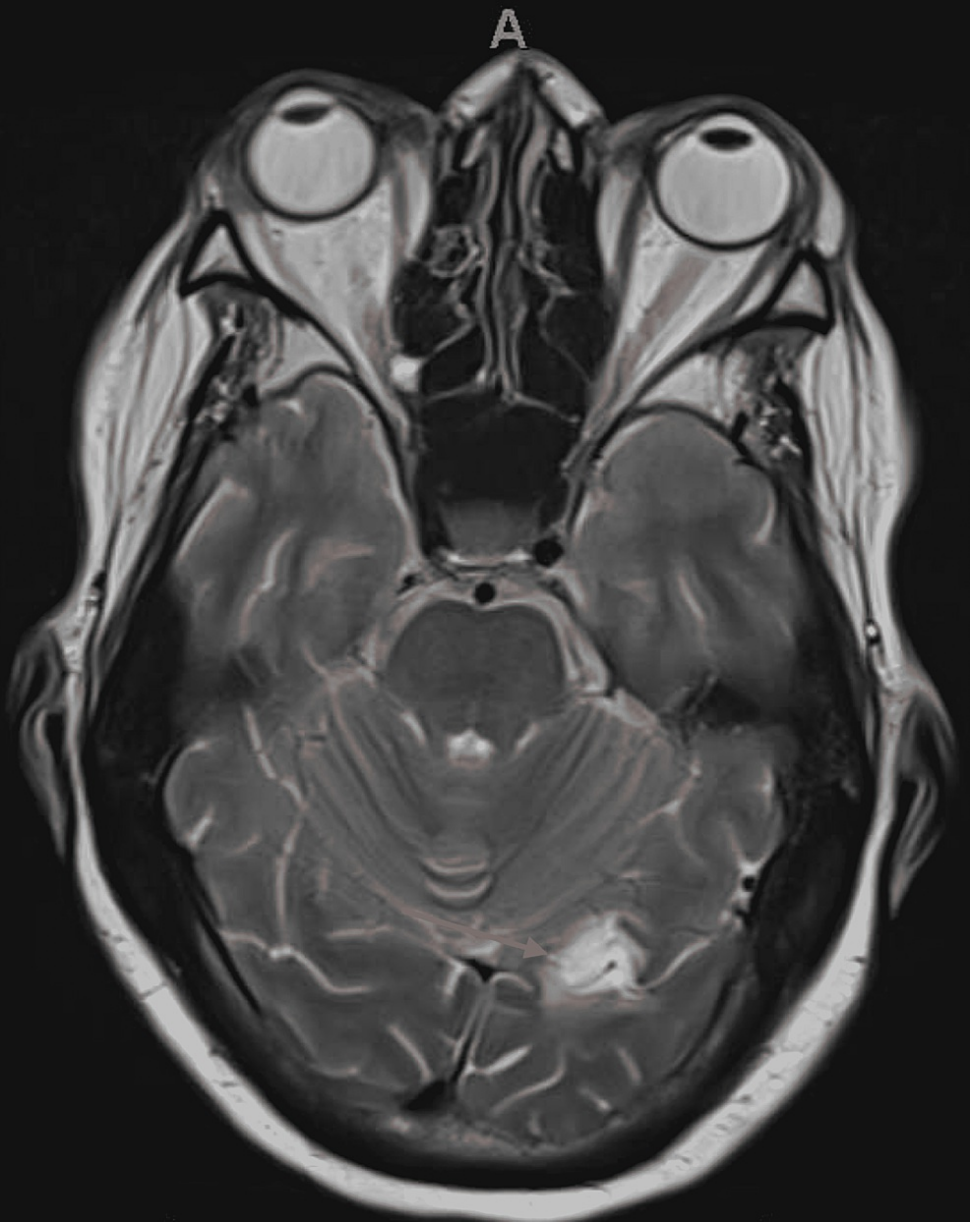
MRI of the brain - image 2 The red arrow is showing the chronic left occipital lobe lesion on this T2-weighted MRI image MRI: magnetic resonance imaging

She was started on metoclopramide for nausea, which did provide mild relief. Gastroenterology was consulted for further evaluation of her persistent nausea. A thorough evaluation was performed, which included esophagogastroduodenoscopy (EGD) and small bowel follow-through, both of which were negative. *Cytomegalovirus* and *Helicobacter pylori *testing was performed and returned negative. Unfortunately, nausea and vomiting continued, and hence a nasogastric tube was placed for nutrition and medication administration. Given the negative gastroenterologic workup, a neurologic cause of the nausea was evaluated. A CT of the head was performed, which did not show any acute abnormality but did show chronic infarctions of the left occipital lobe and right parietal lobe. While a fungal brain abscess was considered, the appearance on imaging was more consistent with previous infarctions. An MRI of the brain was then performed to further characterize the infarctions, which again did not show any acute abnormalities but did redemonstrate the chronic infarctions (Figures 4, 5).

After reassessing her medication list, a voriconazole level was obtained. Her voriconazole trough returned elevated at 11.1 µg/mL (normal therapeutic through: 1.0-6.0 µg/mL). The voriconazole was discontinued and her nausea, vomiting, and weakness slowly improved. She was discharged home with close follow-up. A repeat voriconazole level was obtained about one week after the discontinuation of the voriconazole, which was 6.6 µg/mL. She was then started on isavuconazonium to complete a three-month course of antifungals for her presumed fungal pneumonia, which she was able to complete without any complications. Repeat thoracic CT after the completion of isavuconazonium showed improvement in the cavitary lesion.

## Discussion

Voriconazole is a triazole antifungal that inhibits fungal ergosterol biosynthesis. It works by inhibiting 14-a-lanosterol demethylation, which leads to the accumulation of 14-a-methyl sterols, resulting in the decrease of ergosterol. Ergosterol is an important component of fungal cell wall formation, and hence voriconazole leads to fungal cell wall abnormalities. Voriconazole is indicated for the treatment of invasive aspergillosis as well as resistant infections caused by *S. apiospermum* and *Fusarium* species. Voriconazole is metabolized by the cytochrome P450 enzyme, specifically the CYP2C19 as well as the CYP2C9 and CYP3A4 enzymes [6].

The optimal trough level for voriconazole varies depending on the clinical indication. Some recommendations call for a trough concentration greater than 1 mg/L but less than 4-6 mg/L. Trough levels greater than 4-6 mg/L lead to an increased risk of toxicity. Voriconazole trough levels should be monitored during therapy, with the first level checked in the first two to five days of therapy and further levels should be collected regularly during therapy to ensure stability [7]. It should be noted that the optimal trough concentration for voriconazole is a subject of controversy. One meta-analysis indicated that voriconazole trough levels greater than 3 mg/L were associated with an increased risk of hepatotoxicity; their results also indicated that Asian patients may have a high risk of hepatotoxicity with trough levels of 3 mg/L as compared to non-Asian patients. Furthermore, trough concentrations greater than 4 mg/L were associated with an increased risk of neurotoxicity [8]. The patient in this case had a voriconazole trough level that was significantly elevated at 11.1 mg/L, which certainly places her at an increased risk of toxicity.

Voriconazole has numerous side effects, the common ones being abnormal liver function, visual disturbances, skin rashes, and neurologic disturbances. Nausea, vomiting, and diarrhea are also seen although not as frequently [9].

Fungal infection post-heart transplant is a complication that carries significant morbidity and mortality. The true incidence of invasive fungal infection (IFI) post-heart transplant is not clear but one study did show an incidence of 4.1% within the first year. IFIs are frequently an early complication of a heart transplant, usually occurring within three months of the initial transplant. Numerous factors have been identified that increase the risk of IFI post-transplant, including induction immunosuppression [10]. The patient in this case received induction immunosuppression with basiliximab, which may have placed her at an increased risk for IFI. The use of induction immunosuppression is controversial. About 50% of heart transplant recipients receive induction therapy [11]. Study findings have been varied on the routine use of induction therapy, with some studies showing no significant change in mortality with the use of induction immunosuppression while other studies have shown improved outcomes with routine use of induction immunosuppression. One important consideration regarding the use of induction immunosuppression is the increased risk of infections, including IFI. This increased risk must be weighed with the possibility of improved outcomes with induction immunosuppression.

## Conclusions

IFIs are feared complications of heart transplants, and they are associated with an increased risk of morbidity and mortality. There are numerous risk factors that may increase the likelihood of IFIs post-heart transplant, one of which is induction immunosuppression. While the benefits of induction immunosuppression are a matter of controversy, the risks are better understood and include increased infection risk, including fungal infections. Unfortunately, fungal infections in immunosuppressed patients can be difficult to treat, often requiring long courses of antifungal medications, which carry their own potential risks. Voriconazole is becoming more commonly used for the treatment of IFI due to its broad spectrum of activity. Adequate monitoring of voriconazole levels and subsequent dosing adjustments are imperative for the successful treatment of IFIs while minimizing potential adverse drug reactions. This case highlights the importance of promptly identifying potential adverse drug reactions. Unfortunately, in this case, the delay in identifying an adverse drug reaction led to a potentially unnecessary cholecystectomy, multiple hospitalizations, and a prolonged course of symptoms.
